# Mesenchymal stem cell-mediated suppression of hypertrophic scarring is p53 dependent in a rabbit ear model

**DOI:** 10.1186/scrt526

**Published:** 2014-12-15

**Authors:** Yi-Lun Liu, Wei-Hua Liu, Jin Sun, Tuan-Jie Hou, Yue-Ming Liu, Hai-Rong Liu, Yong-Hui Luo, Ning-Ning Zhao, Ying Tang, Feng-Mei Deng

**Affiliations:** Department of Burns and Plastic Surgery, First Affiliated Hospital of Chengdu Medical College, Chengdu, China; Department of Pathology, Chengdu Medical College, 783 Xindu Avenue, Xindu District, Chengdu, 610500 China; Plastic and Cosmetic Surgery Center, Subei People’s Hospital of Jiangsu Province, Yangzhou, China

## Abstract

**Introduction:**

Mesenchymal stem cells (MSCs) are considered to play important roles in wound repair and tissue remodeling. Hypertrophic scar (HTS) is a cutaneous condition characterized by deposits of excessive amount of collagen after an acute skin injury. However, currently there is little knowledge about the direct relationship between MSCs and HTS.

**Methods:**

The hypertrophic scar model was established on rabbit ears. MSCs were isolated from rabbit femur bone marrow and transplanted through ear artery injection. Hypertrophic scar formation was examined using frozen-section analysis, hematoxylin and eosin (HE) staining, Masson’s trichrome staining, and scar elevation index. The role of p53 in the MSCs-mediated anti-scarring effect was examined by gene knockdown using p53 shRNA.

**Results:**

In this study, MSCs engraftment through ear artery injection significantly inhibited the hypertrophic scarring in a rabbit ear hypertrophic scar model, while this anti-scarring function could be abrogated by p53 gene knockdown in MSCs. Additionally, we found that MSCs down-regulated the expression of TGF-β receptor I (TβRI) and alpha-smooth muscle actin (α-SMA) at both mRNA and protein levels in a paracrine manner, and this down-regulation was rescued by p53 gene knockdown. Moreover, our results showed that MSCs with p53 gene knockdown promoted the proliferation of fibroblasts through increasing nitric oxide (NO) production.

**Conclusions:**

These results suggest that MSCs inhibit the formation of HTS in a p53 dependent manner through at least two mechanisms: inhibition of the transformation of HTS fibroblast to myofibroblast; and inhibition of the proliferation of fibroblasts through inhibition of NO production.

## Introduction

Hypertrophic scar (HTS) is a common complication of burn injury and other soft tissue injuries. Cosmetic and functional impairment caused by HTS remains a great challenge to burn and plastic surgeons [1, 2]. HTS is characterized by the proliferation of a large number of fibroblasts, accumulation of collagen and infiltration of inflammatory cells [3]. Apart from the fibroblasts that have been recognized as one of the driving factors of HTS, mesenchymal stem cells (MSCs) were found to have multiple functions in the formation of HTS [3–5]. MSCs are multipotent cells that can migrate to the wound sites, where they form part of the microenvironment [6–8], improve wound healing and inhibit hypertrophic scarring [9–11]. In addition to the differentiation potential, MSCs can interact with many kinds of cells in the microenvironment through paracrine signaling pathways [12, 13]. Activated MSCs can produce abundant oxidizing agents such as nitric oxide (NO) and cytokines, through which MSCs potently suppress immune responses and influence tumor cell proliferation and phenotype transformation in the tumor microenvironment [14, 15]. However, the mechanisms of the anti-scarring function of MSCs and the interaction between MSCs and HTS fibroblast remain poorly understood.

Pathological scar is considered a tumor-like tissue structure exhibiting an uncontrolled growth manner following wound healing. As one of the most intensively studied tumor-suppressor genes, p53 is also involved in the formation of pathological scar including HTS [16, 17]. An elevated p53 protein level was detected in HTS tissue, compared with normal scar or atrophic white scar [18], but the exact functions of p53 in the scar formation are still not clear.

Recently, studying the roles and mechanisms of stromal cells in tumor formation is a popular field. One study showed that the p53 gene status in tumor-infiltrating MSCs influenced the development of tumor [12]; thus it is an interesting question whether the p53 gene status in MSCs can influence HTS formation. A better understanding of the roles of p53 gene status in the stromal cells would probably provide important insight into HTS pathogenesis. In the present study, we examined the contribution of p53 in MSCs to HTS formation by administering MSCs with or without p53 stable knockdown into rabbit ear HTS models. HTS formation was examined by frozen-section analysis, hematoxylin and eosin (HE) staining and Masson’s trichrome staining, and was evaluated using the scar elevation index (SEI). Our results showed that wild-type MSCs exerted an anti-scarring effect on the HTS model, but p53-deficient MSCs had little influence on the development of HTS. Instead, p53-deficient MSCs resulted in scar recurrence compared with wild-type MSCs. Further study showed that p53 knockdown abrogated the capability of MSCs to inhibit the transformation of HTS fibroblast to myofibroblast. Moreover, p53 deficiency in MSCs resulted in higher NO production, which may promote HTS fibroblast proliferation. Taken together, our study revealed an important role for p53 in MSCs during wound healing and the HTS formation process.

## Methods

### Reagents

Puromycin, *N*^G^-monomethyl-l-arginine acetate salt (L-NMMA) and Griess reagent were from Sigma-Aldrich (St. Louis, MO, USA). Rabbit p53 shRNA lentiviral particles, control shRNA lentiviral particles and rabbit polyclonal antibody against p53 were purchased from Abiocode Bio-Technology (Shanghai, China). Fluorescein isothiocyanate-conjugated antibodies against CD45 and CD90 and phycoerythrin-conjugated antibodies against CD34 and CD105 were from BD Biosciences Pharmingen (San Diego, CA, USA). Dexamethasone, ascorbic acid and β-glycerophosphate were from Sigma-Aldrich Chemicals (St. Louis, MO, USA). MesenCult medium with adipogenic stimulatory supplements was from StemCell Technologies (Vancouver, BC, Canada). The mouse monoclonal antibody against CD31 and the rabbit polyclonal antibodies against transforming growth factor-beta receptor 1 (TβRI) and alpha-smooth muscle actin (α-SMA) were from Santa Cruz Biotechnology (Santa Cruz, CA, USA). Recombinant rabbit interferon gamma and tumor necrosis factor alpha (TNFα) were from eBiosciences (La Jolla, CA, USA).

### Ethics statement, rabbits and cell culture

This study was approved by Animal Experimentation Ethics Committee of the First Afiliated Hospital of Chengdu Medical College, China. All animal treatments were carried out in accordance with Guidelines on Care and Use of Laboratory Animals issued by the Chinese Council on Animal Research and the Guidelines of Animal Care.

New Zealand white rabbits were purchased from Experimental Animal Center of Sichuan Province, with an initial body weight of 2.0 ± 0.3 kg (for the rabbit ear model of hypertrophic scarring). Animals were housed in a regulated environment (22 ± 2°C), with a 12-hour light/dark cycle (light cycle was from 08:00 to 20:00). All animals were fed according to the Specific Pathogen Free Animal Criteria.

MSCs were generated from femur bone marrow using New Zealand white rabbits (1 month old, male) by the holo-bone marrow adherence method. In brief, the bone marrow fluids were extracted under aseptic conditions and transferred into tube with isopyknic Hanks liquid, and centrifuged at 1,000 rpm for 5 minutes at 4°C. After removing supernatant, cells were washed with Hanks liquid and cultured in Dulbecco’s modified Eagle’s medium supplemented with 10% fetal bovine serum, 2 mM glutamine, 100 U/ml penicillin and 100 μg/ml streptomycin (Invitrogen, Carlsbad, CA, USA). Nonadherent cells were removed 8 hours later, and adherent cells were maintained with medium replenishment every 3 days. The cells reached 90% confluence after 14 to 16 days, and were passaged at a split ratio of 1:3 to 5. The cells of passages 4 to 6 were used for experiments. Lineage cell surface markers (CD34, CD45, CD90, and CD105) were detected using flow cytometry (BD Accuri C6; Becton Dickinson San Jose, CA, USA).

HTS fibroblasts were generated from the rabbit ear HTS tissue. In brief, the rabbit ear HTS tissue was cut into 0.5 mm pieces and digested with 0.25 g/l dispase II. The dermal tissue was minced and digested with 30× volume of collagenase I (200 U/ml) at 37°C for 2 hours, followed by centrifugation. The cells were collected and cultured in Dulbecco’s modified Eagle’s medium containing 15% fetal bovine serum at 37°C in 5% carbon dioxide. The culture medium was changed every 3 days. At 80 to 90% confluence, cells were passaged and cultured in Dulbecco’s modified Eagle’s medium containing 10% fetal bovine serum. The cells of passages 4 to 6 were used for further experiments.

The method of isolating engrafted MSCs from scar tissue is the same as for HTS fibroblast isolation [19]. Ten days after isolation, the cells in six-well plates were observed using fluorescent microscope. The cells that show a green fluorescence signal are engrafted MSCs, while the cells without a green fluorescence signal are HTS fibroblasts. The number of fluorescent cells/total cells was calculated in five randomly selected microscopic fields (×100) of each well. Three scar tissues from each group were examined.

### Flow cytometry analysis

Cells were trypsinized, washed with phosphate-buffered saline, and incubated with antibodies against CD34, CD45, CD90 and CD105. Flow cytometry detection was performed with a BD Accuri C6 flow cytometer (Becton Dickinson).

### Osteogenic induction and adipogenic induction

For osteogenic induction, cells with 70% confluence were incubated in osteogenic medium supplemented with 10^−7^ M dexamethasone, 0.2 mM ascorbic acid and 10 mM β-glycerophosphate. The medium was changed twice a week. Cell colonies displayed bone-like nodular aggregates of matrix mineralization after 14 days in differentiation medium. The cells were stained with alizarin red. For adipogenic induction, MesenCult medium with adipogenic stimulatory supplements was used to treat the cells. The medium was changed every 3 to 4 days. The cells were stained with oil red after culturing for 21 days.

### Lentivirus transduction

Rabbit bone marrow MSCs were transduced by lentiviral particles containing rabbit p53 shRNA or control shRNA for 24 hours, and then subjected to puromycin (3 μg/ml) selection. Stable clones were harvested 2 weeks later, and passaged with normal media.

### Western blot

Cells were lysed in lysis buffer containing phenylmethylsulfonyl fluoride, leupeptin and other protease inhibitors for 1 hour on ice. Protein samples were separated on sodium dodecyl sulfate–polyacrylamide gels, then transferred onto polyvinylidene difluoride membranes, and incubated with corresponding primary antibodies at 4°C overnight. After washing, membrane was incubated with secondary antibodies, and subjected to chemiluminescent detection according to the manufacturer’s instructions.

### 3-(4,5-dimethylthiazol-2-yl)-5(3-carboxymethonyphenol)-2-(4-sulfophenyl)-2H-tetrazolium (MTS) assays

Cells were seeded into flat-bottomed 96-well culture plates at 5,000 cells/well, and grew for 48 hours. Cells were labeled with the VisionBlue reagent and detected by Synergy 2 multidetection microplate reader (BioTek, Winooski, VT, USA) according to the manufacturer’s instruction.

### Colony formation assay

Cells were seeded at 2,000 cells/well onto six-well culture plates and allowed to grow for 12 days before fixation with methanol and staining with crystal violet (0.5% solution).

### Rabbit ear model of hypertrophic scarring, grouping and MSC engraftment

The rabbit ear model of hypertrophic scarring was established according to the procedure previously described by Kryger and colleagues with a minor modification [20]. In brief, rabbits were intravenously anesthetized with sodium pentobarbital 30 mg/kg. Under aseptic operation conditions, four round wounds each with a diameter of 9 mm were created down to the bare cartilage on the ventral surface of each ear along the long axis. The epidermis, dermis and perichondrium in each wound were thoroughly removed, and the wounds were covered with sterile gauze for 1 day. After this operation, the rabbits were provided with conventional anti-inflammatory treatment. Three weeks after operation, the healed wound surface showed an obvious protrusion, an indicator of HTS formation. Five weeks post operation, the formation of HTS reached the peak level.

Twelve rabbits were divided into three groups with age and gender matched: control group; control shRNA MSC group; and p53 shRNA MSC group. Three days after the model operation, 1 × 10^5^ cells of control shRNA MSCs or p53 shRNA MSCs (in 1 ml phosphate-buffered saline) were injected into the rabbits via ear artery once every 2 days up to eight times. Phosphate-buffered saline was used in the control group.

### General observation and histological analysis

General observations including growth status, color, texture and thickness of HTS were recorded by photographic imaging at designated time points. In addition, the height of HTS was measured.

Full-thickness biopsies of the wound-repair bed and surrounding tissue were obtained at 3, 4 and 5 weeks after operation. The tissues were subjected to frozen-section analysis. Paraffin sections were subsequently subjected to HE staining, and Masson’s trichrome staining using Masson’s Trichrome Stain Kit (KeyGen Biotech Co., Nanjing, China) following the manufacturer’s instructions. In brief, the sections were stained in Weigert’s iron hematoxylin working solution for 10 minutes and in Biebrich scarlet-acid fuchsin solution for 5 minutes, and then placed in 1% phosphomolybdic–phosphotungstic acid solution for 5 minutes. The sections were then stained in aniline blue solution for 5 minutes, and rinsed in 1% acetic acid solution for 2 minutes. Collagen fibers were stained blue. Keratin and muscle fibers were stained red. Cell cytoplasm and nuclei were stained light pink and dark brown, respectively. In each section of Masson’s trichrome staining, three high-power fields in the dermis of wound area or unwounded normal tissue, inferiorly by the ear cartilage and superiorly by the epithelial basement membrane, were randomly selected and photographed. The integrated optical density analysis of Masson’s trichrome staining was performed by Image-Pro Plus 6.0 software (Media Cybernetics, Silver Springs, MD, USA) [21].

The SEI, which measures the ratio of total scar connective tissue area to the area of underlying dermis, was evaluated using Image-Pro Plus 6.0 software. The height of the underlying dermis was determined based on the height of the adjacent unwounded dermis. An SEI of 1 indicated no newly formed hypertrophied dermis, whereas SEI >1 denoted HTS formation [22]. CD31 in the scar tissues was detected by immunohistochemistry using monoclonal anti-CD31 antibody (1:100).

### Real-time PCR

Total RNA was isolated using RNAprep pure Cell/Bacteria Kit (Tiangen Biotech, Beijing, China), and first-strand cDNA synthesis was performed using the 1st cDNA Synthesis Kit with oligo(dT)_15_ (Tiangen Biotech). The mRNA levels were measured by real-time PCR using SYBR Green Master Mix (Roche Diagnostics, Indianapolis, IN, USA). The total amount of mRNA was normalized to endogenous β-actin mRNA.

Sequences of PCR primer pairs were as follows: TβRI, forward primer 5′-TGTTGGTACCCAAGGAAAGC-3′ and reverse primer 5′-CACTCTGTGGTTTGGAGCAA-3′; α-SMA, forward primer 5′-CAGGGAGTAATGGTTGGAAT-3′ and reverse primer 5′-TCTCAAACATAATCTGGGTCA-3′; inducible nitric oxide synthase (iNOS), forward primer 5′-CAGCTGGGCTCAACAAACCTT-3′ and reverse primer 5′-CATTGGAATGGAAGCGTATCG-3′; and β-actin, forward primer 5′-GCTATTTGGCGCTGGACTT-3′, and reverse primer 5′-GCGGCTCGTAGCTCTTCTC-3′.

### Detection of nitric oxide

NO was detected using a modified Griess method. Briefly, all NO_3_^−^ was converted into NO_2_^−^ by nitrate reductase, and total NO_2_^−^ was detected by the Griess reaction [23].

### Statistical analysis

SPSS Version 13.0 for Windows (SPSS Inc., Chicago, IL, USA) was used for data analysis. Data were presented as the mean ± standard error of the mean. Statistical significance was assessed by one-way analysis of variance. Significance was set at *P* <0.05.

## Results

### Establishment of the p53 stable knockdown cell line of rabbit MSCs

**Figure 1:**
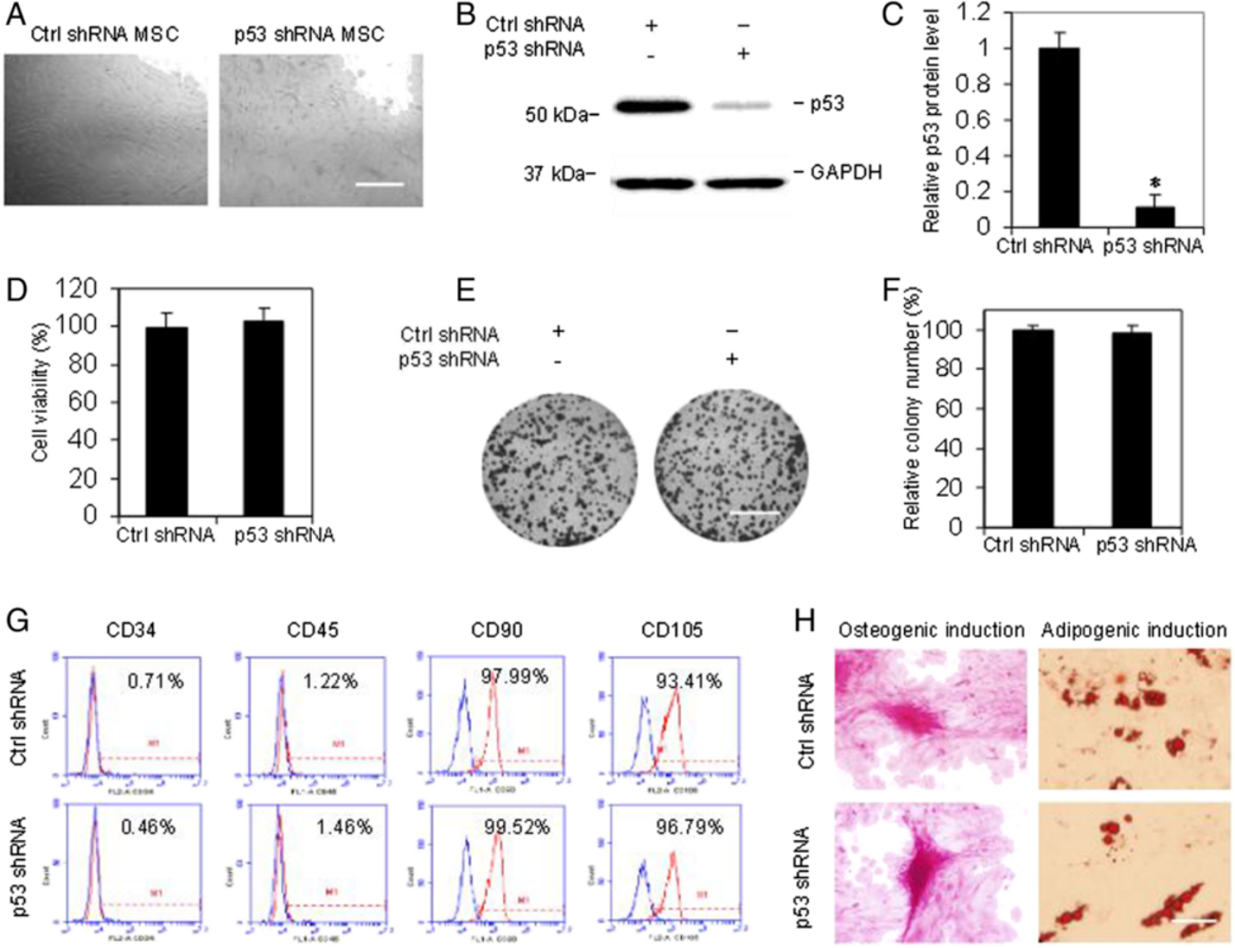
**Establishment of p53 stable knockdown in rabbit mesenchymal stem cells.** Passage 4 of mesenchymal stem cells (MSCs) generated from femur bone marrow was transduced with lentiviral particles containing p53 shRNA or control (Ctrl) shRNA. **(A)** Stable clones were harvested after puromycin selection, and cells of passages 3 to 9 were used for the following tests. Scale bar, 500 μm. **(B)** Representative western blot image of p53 in MSCs. **(C)** Quantification of p53 protein level in western blot by densitometry. Glyceraldehyde 3-phosphate dehydrogenase (GAPDH) used as internal control (*n* = 3, *P <0.01 vs. control group). **(D)** 3-(4,5-dimethylthiazol-2-yl)-5(3-carboxymethonyphenol)-2-(4-sulfophenyl)-2H-tetrazolium (MTS) assay showed no significant difference in cell viability between the two groups. *P* >0.05. **(E)** Representative images of cell colony formation. Viable cells (2,000 cells/well in six-well plates) were allowed to grow for 12 days before being fixed with methanol and stained with crystal violet. Scale bar, 1 cm. **(F)** Quantification of the number of colony formation in p53 shRNA and control shRNA groups. *P* >0.05. **(G)** Flow cytometry analysis of cell surface markers. Blue line, negative control. **(H)** Differentiation capacity of the cells. Both control shRNA-transduced and p53 shRNA-transduced MSCs differentiated into mineralizing cells stained with alizarin red, or adipocytes stained with oil red. Scale bar, 200 μm. Data presented as mean ± standard error of the mean, *n* = 3 individual experiments.

Passage 4 of MSCs was transduced with lentiviral particles containing p53 shRNA or control shRNA, each with a green fluorescent protein label. After transduction, cells were selected with puromycin, then passaged for further use (Figure 1A). p53 knockdown was successfully achieved as shown by western blot. The expression of p53 protein decreased more than 80% (Figure 1B,C). 3-(4,5-dimethylthiazol-2-yl)-5(3-carboxymethonyphenol)-2-(4-sulfophenyl)-2H-tetrazolium (MTS) and colony formation assays were performed to test the cell viability and colony formation capacity of p53 shRNA-transduced or control shRNA-transduced cells. There was no significant difference between the two groups on 3-(4,5-dimethylthiazol-2-yl)-5(3-carboxymethonyphenol)-2-(4-sulfophenyl)-2H-tetrazolium (MTS) and colony formation (Figure 1D,E,F). CD90 and CD105 were highly expressed in both p53 shRNA-transduced and control shRNA-transduced MSCs. CD34 and CD45 expressions were very low in the above cells (Figure 1G). Both kinds of cells would differentiate into mineralizing cells stained by alizarin red after osteogenic induction or adipocytes stained by oil red after adipogenic induction (Figure 1H). These results suggested that p53 shRNA-transduced and control shRNA-transduced MSCs maintained the common features of MSCs.

### Exogenous MSCs migrated into rabbit ear hypertrophic scar tissue by transplantation via ear artery

**Figure 2:**
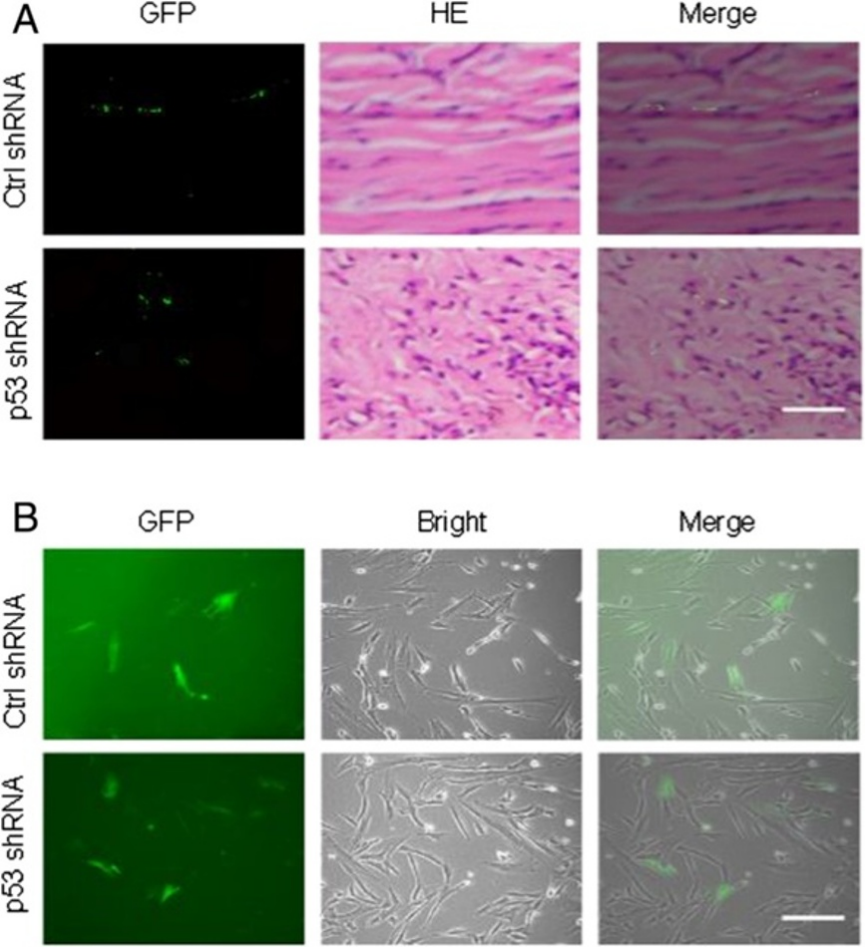
**Mesenchymal stem cells with or without p53 stable knockdown, both with green fluorescent protein label, were injected via ear artery during the wound healing process of the rabbit ear hypertrophic scar model, and migrated into the scar tissue. (A)** Frozen-section analysis and hematoxylin and eosin (HE) staining of scars 4 weeks after model operation. The green fluorescence in the frozen sections indicated the presence of the transplanted mesenchymal stem cells (MSCs) that migrated into the scar tissue. Scale bar, 200 μm. **(B)** Representative cultured MSCs that were isolated from the scar tissue. Scale bar, 200 μm. Ctrl, control; GFP, green fluorescent protein.

Endogenous MSCs are known to be recruited to the sites of damaged tissue, while exogenous MSCs have also been shown to migrate to target tissue by transplantation via associated artery or by bone marrow transplantation [12, 24]. In this study, to examine whether exogenous MSCs were recruited into the HTS tissue after transplantation via ear artery, MSCs with or without p53 stable knockdown, both with a green fluorescent protein label, were injected into the rabbit ear via the ear artery during the wound healing process in the rabbit ear HTS model. Scars were excised and subjected to frozen-section analysis and HE staining 4 weeks after model operation. The green fluorescence in the frozen sections indicated the presence of the transplanted MSCs that migrated into the scar tissue (Figure 2A). MSCs were also isolated from the scar tissue and cultured in the media. A fraction of the scar tissue-infiltrated MSCs expressed green fluorescent protein in both p53 shRNA-transduced and control shRNA-transduced cells (14.8% ± 0.9, and 15.3 ± 0.7 respectively) (Figure 2B).

### p53 is essential for MSCs to prevent the progression of hypertrophic scar formation in the rabbit ear model

**Figure 3:**
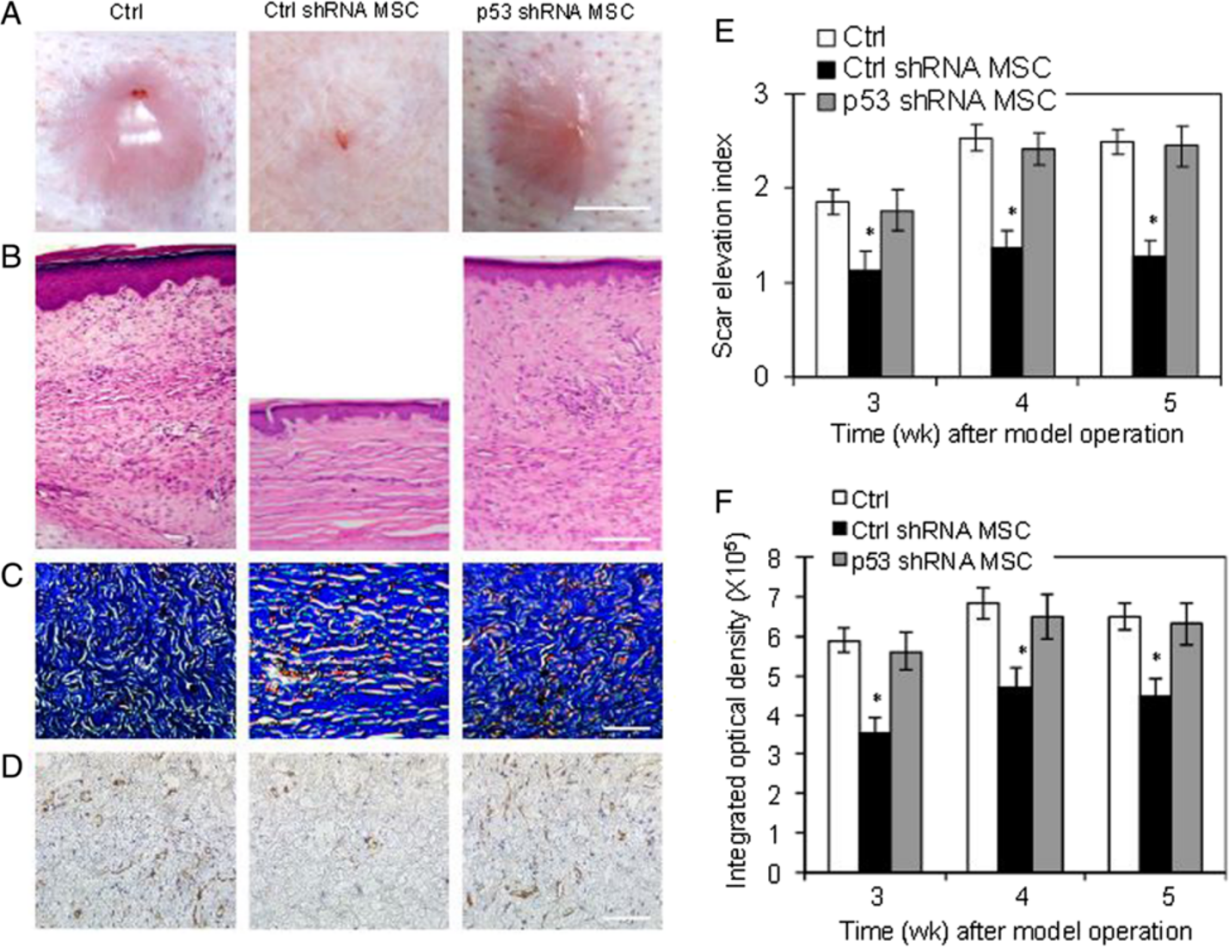
**Role of p53 in mesenchymal stem cell-induced prevention of hypertrophic scar in the rabbit ear model.** Representative **(A)** general observations (scale bar, 5 mm), **(B)** hematoxylin and eosin staining (scale bar, 500 μm), **(C)** Masson’s trichrome staining (scale bar, 200 μm), and **(D)** immunohistochemistry of CD31 (scale bar, 200 μm) of scar sections 4 weeks after model operation from three individual experiments. **(E)** Scar elevation index (SEI). **(F)** Integrated optical density analysis of Masson’s trichrome staining. Data expressed as mean ± standard error of the mean, *n* = 6. *P <0.05 vs. control (Ctrl) group and p53 shRNA mesenchymal stem cell (MSC) group.

The control group without MSC treatment showed successful HTS formation after operation characterized by obvious hyperplasia, collagen fiber accumulation and abundant blood vessels, according to the general observations and histological analysis (Figure 3A,B,C,D, first panel). MSCs transduced with control shRNA inhibited the HTS formation (Figure 3A,B). The SEI was significantly reduced in control shRNA-transduced MSCs group at 3, 4 and 5 weeks after model operation compared with control group without MSCs and the p53 shRNA-transduced MSC group (Figure 3E). The control shRNA-transduced MSC group showed less collagen deposition, indicated by lighter Masson’s trichrome staining (Figure 3C,F) and less CD31-positive blood vessels (Figure 3D) by immunohistochemistry compared with the control group without MSCs and the p53 shRNA-transduced MSC group. However, there was no significant difference in the SEI and histological results between the p53 shRNA-transduced MSC group and the normal control group. Taken together, the results indicated that MSCs significantly inhibited the development of HTS and that p53 knockdown abrogated the effects of MSCs on HTS formation, suggesting that p53 may be essential for MSCs to prevent HTS formation.

### TβRI and α-SMA in fibroblast were downregulated by MSCs in a p53-dependent manner

**Figure 4:**
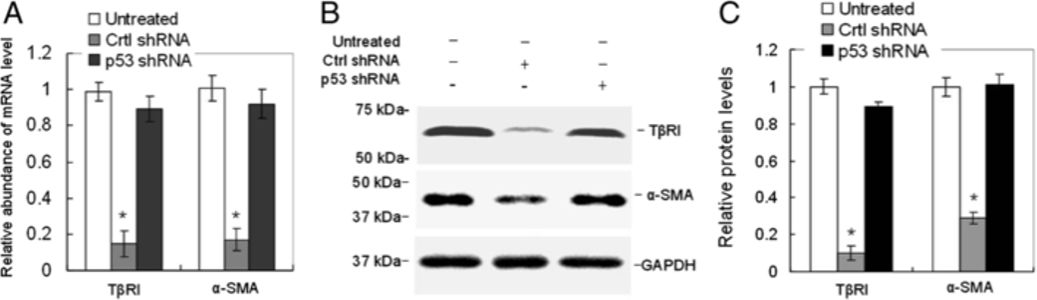
**TβRI and α-SMA of fibroblasts were downregulated by mesenchymal stem cells in a p53-dependent manner.** Fibroblasts derived from rabbit ear hypertrophic scar were treated with or without conditioned medium from control shRNA mesenchymal stem cells (MSCs) or p53 shRNA MSCs for 48 hours. The **(A)** mRNA and **(B,C)** protein levels of TβRI and α-SMA were determined by real-time PCR and western blot. *n* = 3, *P <0.05 vs. untreated group, p53 shRNA group. Data shown are representative of three individual experiments. α-SMA, alpha smooth muscle actin; GAPDH, glyceraldehyde 3-phosphate dehydrogenase; TβRI, transforming growth factor-beta receptor 1.

Transformation of fibroblast to myofibroblast is an important step in HTS development. During the differentiation, fibroblastic cells lose their migratory phenotype and become sessile. Previous studies have shown that the differentiation of scar fibroblast relies on transforming growth factor-beta 1 (TGF-β1) and α-SMA [25]. The TβRI could recognize and bind to TGF-β1 complex in the fibroblasts, and activate the following signaling pathways. We found that conditioned medium from control shRNA-transduced MSCs significantly downregulated TβRI and α-SMA in both mRNA (Figure 4A) and protein (Figure 4B,C) levels, while the conditioned medium from p53 shRNA-transduced MSCs did not affect the expression of TβRI and α-SMA. Our results indicated that the transformation of fibroblast to myofibroblast could be inhibited by MSCs through paracrine in a p53-dependent manner.

### Elevated level of nitric oxide produced by p53-deficient MSCs may be responsible for the promotion of HTS fibroblast proliferation

**Figure 5:**
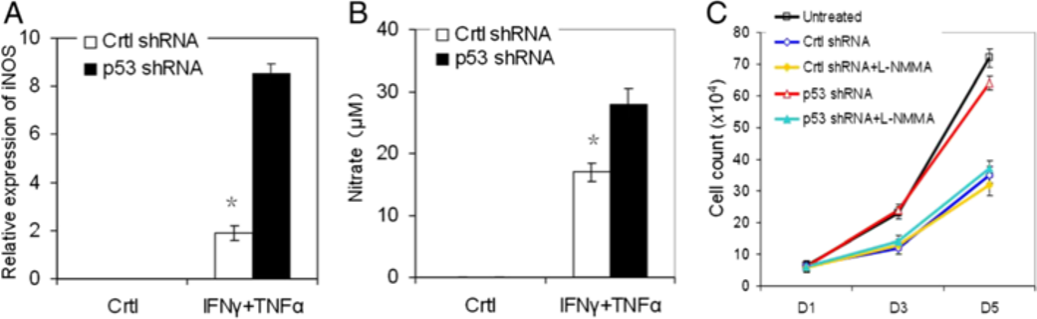
**Elevated nitric oxide level produced by mesenchymal stem cells with p53 deficiency may be responsible for the proliferation promotion of hypotrophic scar fibroblasts.** Mesenchymal stem cells (MSCs) with or without p53 knockdown were treated with or without interferon gamma (IFNγ) and tumor necrosis factor alpha (TNFα) (10 ng/ml each) for 24 hours. **(A)** Inducible nitric oxide synthase (iNOS) mRNA was measured by real-time PCR. **(B)** Total nitrate was measured by Griess. **(C)** Conditioned medium from MSCs treated with IFNγ and TNFα for 24 hours was added to cultured hypertrophic scar fibroblasts with or without treatment with 1 mM *N* ^G^-monomethyl-l-arginine acetate salt (L-NMMA). Cells were trypsinized and counted 48 hours later on the indicated days. *n* = 3, *P <0.05 vs. p53 shRNA group. Data shown are representative of three individual experiments. Ctrl, control.

It was reported that p53 could decrease iNOS expression in certain murine fibroblasts and MSCs by downregulating iNOS promoter activity [26]. After treatment with interferon gamma and TNFα, iNOS mRNA expression in p53 knockdown MSCs was significantly increased compared with control shRNA-transduced MSCs (Figure 5A). NO production was enhanced in p53 knockdown MSCs compared with control shRNA-transduced MSCs (Figure 5B). To examine the roles of NO in the proliferation of HTS fibroblasts, L-NMMA – an iNOS inhibitor – was used to manipulate the NO level in the conditioned medium from MSCs with or without p53 knockdown. L-NMMA significantly ablated the promotion of fibroblast proliferation caused by the conditioned medium from p53 knockdown MSCs (Figure 5C). These results suggested that the elevated production of iNOS and NO by p53 deficiency MSCs may be responsible for the promotion of HTS fibroblast proliferation.

## Discussion

The formation of HTS is considered a result of aberrations of wound healing following any injury to the deep dermis, including burn injury, lacerations, abrasions, surgeries, and so on. Any factor that delays wound healing would increase the chance of hypertrophic scarring. On the contrary, if the process of wound healing is accelerated, the occurrence or degree of scarring would be reduced [2, 3]. As known, the time to heal is the most important factor to predict the development of HTS in a burn wound. If healing occurred between 14 and 21 days, only one-third of anatomical sites became hypertrophic. However, 78% of the sites developed HTS if the burn wound healed after 21 days. The time to heal could influence many other factors involved in HTS formation, including transformation of the wound clot into granulation tissue, which requires a delicate balance between extracellular matrix protein deposition and degradation [27]. Stem cells have been regarded as a potentially useful agent for promoting wound healing. Specifically, bone marrow MSCs were shown to improve wound healing in several studies [4, 5]. Human bone marrow MSCs prevented HTS formation in the rabbit ear HTS model via inflammatory regulation and secretion of an anti-inflammatory protein, TNFα-stimulated gene/protein 6 [28]. The immune-modulating properties, especially the immune-suppression effect, of MSCs have made it widely applicable, although the mechanisms remain unclear. Our current study shows that transplantation of MSCs via the rabbit ear artery is an effective approach for MSC engraftment and migration in the rabbit HTS model. Furthermore, MSCs engraftment significantly reduced the formation of rabbit ear HTS, suggesting that MSCs may have potential clinical applications in modulating the process of wound healing. In addition, we also found less infiltrated inflammatory cells in the scar tissue with HE staining in the control shRNA-transduced MSC group. Although we focus on p53 in the present study, further investigation about inflammatory modulation of MSCs would be performed in the future.

The aberration of gene expression in HTS has been well studied, but the most critical genes in HTS progression remain undefined [29–31]. In the HTS tissue, p53 protein was reported to have a higher level compared with normal dermis tissue [18], but the roles of p53 in HTS have not been fully investigated, and little is known about p53 gene status in MSCs in the HTS tissue. Investigators recently found that MSCs could interact with other types of cells in the microenvironment, resulting in gene expression or even the phenotype changes of those cells; for example, the epithelial–mesenchymal transition of tumor cells [32–35]. Interestingly, the gene status of p53 in the MSCs has been reported to play an essential role in tumor development [12]. We therefore hypothesized that the p53 in MSCs plays an important role in wound healing and then the development of HTS. To test this hypothesis, we established MSCs with stable p53 knockdown, and transplanted them into the rabbit ear HTS model. We found that the engraftment of p53-deficient MSCs can hardly inhibit the scar formation, suggesting that p53 plays an essential role in preventing HTS formation by MSCs. According to this result, the higher expression level of p53 in the HTS tissue as previously reported may be the outcome of self-regulation of anti-scarring in the microenvironment.

Central to the formation of HTS tissue is the transformation of HTS fibroblast to myofibroblast [36], the latter being responsible for the excessive deposition and irreversible remodeling of the extracellular matrix [37]. The TGF-β1 signaling pathway and α-SMA are involved in the phenotype transformation [38, 39]. Our results showed that wild-type MSCs downregulated the expression of TβRI and α-SMA at both the mRNA and protein levels in a paracrine manner, while the p53-deficient MSCs did not affect the expression of these two genes. These data suggest that the inhibition of fibroblast transformation to myofibroblast may account for the anti-scarring outcome by MSC engraftment, in a p53-dependent manner. However, the mechanisms of MSCs regulating the expression of TβRI and α-SMA of fibroblasts are still unclear. Further investigations on these mechanisms will be helpful for understanding the role of MSCs in HTS formation.

The roles of oxidative/nitrosative stress in cell proliferation are controversial [40–42]. Our previous studies showed that the oxidization level in HTS tissue is elevated compared with normal tissue [43]. In this study, we used interferon gamma and TNFα as stimulation for MSCs to produce NO before collecting conditioned medium. Our data suggest that the elevated level of NO produced by MSCs with p53 knockdown is involved in the proliferation promotion of HTS fibroblasts, and MSCs could inhibit the transformation of HTS fibroblast to myofibroblast in a p53-dependent manner.

## Conclusions

The results in the current study demonstrated that wild-type MSC engraftment could inhibit the formation of HTS, in a p53-dependent manner. This effect might be achieved through downregulating TβRI and α-SMA gene expression, and inhibiting the transformation of HTS fibroblast to myofibroblast through reducing NO production.

## Abbreviations

HE: hematoxylin and eosin
HTS: hypertrophic scar
iNOS: inducible nitric oxide synthase
L-NMMA: *N*^G^-monomethyl-l-arginine acetate salt
MSC: mesenchymal stem cell
NO: nitric oxide
SEI: scar elevation index
α-SMA: alpha-smooth muscle actin
TβRI: transforming growth factor-beta receptor 1
TGF-β1: transforming growth factor-beta 1
TNFα: tumor necrosis factor alpha.
